# Sex and Age Differences in Mortality in Southern China, 2004–2010

**DOI:** 10.3390/ijerph120707886

**Published:** 2015-07-10

**Authors:** Leibin Yu, Xinqin Lin, Haiyan Liu, Jian Shi, Quanxing Nong, Hongyang Tang, Zongfu Mao

**Affiliations:** 1 School of Public Health, Wuhan University, Wuhan 430072, China; E-Mail: yuleibin_submit@163.com; 2 Nanning Municipal Center for Disease Control and Prevention, Nanning 530028, China; E-Mails: linxinqin_submit@163.com (X.L.); liuhaiyan_submit@163.com (H.L.); shijian_submit@163.com (J.S.); nongquan_submit@163.com (Q.N.); tanghy_submit@163.com (H.T.)

**Keywords:** mortality, sex and age differences, southern China

## Abstract

The purpose of this study was to describe the mortality patterns in the southern provinces of China, and to provide epidemiologic data on sex and age differences of death outcomes. Reliable mortality and population data from January 2004 to December 2010 were obtained from 12 Disease Surveillance Point (DSP) sites in four provinces of China. Death data from all causes and respiratory disease, chronic obstructive pulmonary disease (COPD), pneumonia and influenza, circulatory disease, and ischemic heart disease, were stratified by year, month of death occurrence and sex, seven age groups, and summarized by descriptive statistics. The mean annual mortality rates of the selected 12 DSP sites in the southernmost provinces of China were 543.9 (range: 423.9–593.6) deaths per 100,000 population. The death rates show that noted sex differences were higher in the male population for all-cause, COPD and circulatory diseases. Pneumonia and influenza death rates present a different sex- and age-related distribution, with higher rates in male aged 65–74 years; whereas the death rates were opposite in elderly aged ≥75 years, and relatively higher in young children. This study had practical implications for recommending target groups for public health interventions.

## 1. Introduction

Calculating the cause-specific death rates by sex and age is the first step to define the disease burden of a population, which is essential for a government to support the development of public health policy. Also it plays a key role in the estimation of the direct and indirect burden of infectious diseases [1]. A typical case is influenza, for which laboratory testing and confirmation are not usually carried out in routine clinical practice, especially in the developing countries. Indirect burden of influenza, including excess hospitalization and mortality, has been estimated for decades using regression models that are combined with disease outcomes with or without influenza virological surveillance data. China has established vital registration systems to provide basic information on number, rates, and ranks of cause-specific deaths among the Chinese population [2]. Additionally, using mortality data of specific categories of respiratory or circulatory diseases, we could assess the impact of seasonal and pandemic influenza in different regions of China, for example, the northern temperate and southern subtropical regions [3,4].

Southeast Asian areas, including southern China with subtropical and tropical climate, are usually believed to be the epicenters of novel influenza viruses and future pandemics. A recent study had identified three epidemiological regions in China, characterized by distinct influenza seasonality, representing its more complex patterns in provinces at intermediate latitudes (within 27.4° N–31.3° N) and in provinces in southernmost (latitude < 27° N) [5]. In addition, interestingly, more recent studies revealed sex-specific risks and differential disease outcomes in patients with viral infections (influenza, respiratory syncytial virus, and adenovirus), bacterial infections (Tuberculosis, Group A streptococcal pharyngitis, and pneumococcal disease), and parasitic infections (Leishmaniasis, Trypanosomiasis, and Chagas disease) [6,7].

In the present study, based on a timely population-based vital statistics registry system, Disease Surveillance Points (DSP), we aimed to describe the mortality patterns of all-cause and specific categories of respiratory and circulatory diseases in the southernmost provinces of China, and to provide epidemiologic data on sex and age differences of these death outcomes.

## 2. Materials and Methods

### 2.1. Source of Mortality and Population Data

**Figure 1:**
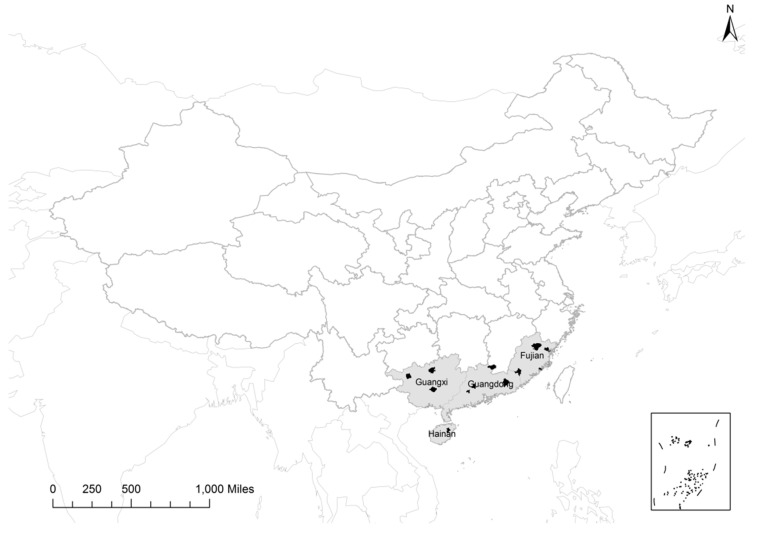
Distribution map of selected mortality surveillance sites in Southern China.

The source of mortality data we used in this study was the national representative sample-based DSP system, which was designed to collect long-term basic information of births, causes of death, and incidence of the 35 notifiable infectious diseases among the Chinese population. The system was previously established by the Chinese Academy of Preventive Medicine, the precursor of the Chinese Center for Disease Control and Prevention (China CDC) in 1990. It included 145 sites, which were at either township (“Xiang”) level in rural areas or neighborhood (“Jiedao”) level within urban cities scattered over the 31 provinces of China at early stage [2]. In 2003, the DSP sites were re-sampled based on counties in rural areas or districts within cities using a multistage cluster probability sampling method, aiming to be more representative of national population. All counties (representing rural area) and districts (representing urban area) within 31 provinces were divided into three strata of eastern, central and western region; then, rural (counties) and urban (districts) areas were divided into three strata based on per-capita gross domestic product and the proportion of non-agricultural population separately; population size was used to divide into another three strata in the third stage. In summary, the counties or districts were divided into 27 strata respectively. Currently, the system includes 161 sites (counties or districts) in 31 provinces covering a population of 72.1 million. Therefore, the DSP sites were selected to be representative for the whole country of China; it could reflect regional distributions of population in eastern, central, and western China, various economic levels in urban and rural areas, and basic demographic characteristics by age and sex [8]. The DSP system covers 19 sites in four provinces of Guangdong, Guangxi, Fujian, and Hainan. In this study, we found that 12 participating sites had the most reliable mortality data covering a population of 6.36 million, or 3.47% of the total population of the four provinces in 2010 (Figure 1), which were based on the following inclusion criteria: Average all-cause mortality rate during the study period ≥4.5‰ with mortality rate in any given year ≥3‰, and average percentage of deaths with unknown-causes <5% throughout the study period, while with an annual percentage <10% in any given year [9]. We included mortality data from the 12 DSP sites from January 2004, when vital statistics registries became established with well-defined population denominators, till the end of 2010. Population size denominators in these sites were obtained from the annual mid-year population statistics of the National Bureau of Statistics of China.

The procedure for vital registration was conducted following the DSP surveillance guidelines [10]. In urban areas, about half of the people died in hospitals at different levels, so their vital registration would adhere to standard protocols [11]. For deaths which occurred at home or other places, the attending physicians would write medical certificates to provide causes of death, according to the registration process in the protocols. In rural areas, only a few deaths occurred at township hospitals or other secondary or tertiary hospitals in the neighboring areas, and almost 80% of people died at home or other non-hospital places [11]. Even for those who died outside hospitals, there was usually clinical evidencs available from their latest visits to township or other hospitals when seeking medical advice. The procedures for the collection and compilation of cause-of-death data were described in a previous study [2].

A web-based online data management system was established by China CDC to collect individual death certificates each week, including the demographic information and the underlying causes of death coded based on the International Classification of Diseases, 10th Revision [ICD-10] in all surveillance sites [12]. In 2006, by conducting a national repeated retrospective sampling survey of causes of death between 2004 and 2005, the quality of reported mortality data in the expanded DSP system was checked [9]. In that survey, 868,484 deaths were recorded based on confirming the name list of all deaths, checking the death-cause by reviewing certificates of deaths and household inquiry, coding by qualified professionals, verifying the quality of the certificate of deaths, and so-called verbal autopsy procedures with an international tool validated in multi-sites were used to characterize and check misclassification of the deaths without hospital diagnosis [13]. This large-scale comprehensive survey represented the real situation of deaths, therefore, mortality data we used in this study from the DSP system in 2004 and 2005 were verified by the 2006 survey mentioned above. Independent re-surveys, based on “capture-mark-recapture” methods, also are used to estimate the completeness of registration of the DSP system. The recent under-reporting survey of mortality registration was conducted in 2009. However, the under-reporting survey could not provide site and cause specific rates, so the mortality data from 2006 were not adjusted.

### 2.2. Statistical Analysis

Given that respiratory and circulatory diseases are important causes of deaths in China, per our previous studies, we obtained separate data of deaths from all-cause and of deaths caused by respiratory disease (ICD-10, codes J00–J99), chronic obstructive pulmonary disease (COPD, codes J40–J47), pneumonia and influenza (codes J10–J18), circulatory disease (codes I00–I99), and ischemic heart disease (codes I20–I25) [3,4]. Mortality data were stratified by year, month of death occurrence and sex, age groups (0–11 months, 1 year, 2–4 years, 5–14 years, 15–64 years, 65–74 years, and ≥75 years). Descriptive statistics was used to summarize the cause-specific mortality rates by sex, age group, and to analyze their temporal trends using monthly data within the study period. Age-standardized death rates were also calculated to compare year-to-year level. Ninety five percent confidential interval (CI) were provided by age-group and sex to test the difference of deaths rates.

## 3. Results

### 3.1. Annual Mortality Rates

Between 2004 and 2010, the mean annual mortality rates of the selected 12 DSP sites in the four southernmost provinces of China, were 543.9 (range: 423.9–593.6) deaths per 100,000 population. The coded causes of 17.8% and 34.1% of all deaths were respiratory and circulatory disease (Table 1). Among the respiratory disease deaths, 79.9% were COPD, and 15.7% were pneumonia and influenza deaths, while among circulatory diseases, 25.0% were ischemic heart disease. Death rates were relatively lower in 2006–2007, and rates in 2009–2010 were roughly equal to those in 2004–2005 which was verified by the 2006 survey.

**Table 1:** Annual death rates (per 100,000 people) by all-cause death in Southern China, 2004–2010.

Year	Population (Thousand)	Number of Deaths/100,000 People (% of All Cause Deaths)					
		All Causes	Respiratory Diseases	COPD	Pneumonia and Influenza	Circulatory Diseases	Ischemic Heart Disease
2004	6449	578.2	107.6 (18.6)	88.4 (15.3)	14.3 (2.5)	188.1 (32.5)	48.6 (8.4)
2005	6503	580.5	112.0 (19.3)	91.2 (15.7)	15.5 (2.7)	186.9 (32.2)	47.3 (8.1)
2006	6529	423.9	74.6 (17.6)	58.2 (13.7)	12.3 (2.9)	136.3 (32.2)	28.5 (6.7)
2007	6571	506.2	88.4 (17.5)	69.2 (13.7)	15.2 (3.0)	169.2 (33.4)	39.9 (7.9)
2008	6601	548.6	96.0 (17.5)	75.7 (13.8)	16.9 (3.1)	192.5 (35.1)	47.5 (8.7)
2009	6294	593.6	102.1 (17.2)	82.3 (13.9)	16.0 (2.7)	215.6 (36.3)	55.4 (9.3)
2010	6357	580.0	96.1 (16.6)	75.6 (13.0)	16.5 (2.8)	212.0 (36.5)	57.4 (9.9)
Mean	6472	543.9	96.6 (17.8)	77.2 (14.2)	15.2 (2.8)	185.5 (34.1)	46.3 (8.5)
**Year**	**Population (Thousand)**	**Age-Standardized Deaths Rates/100,000 People (% of All Cause Deaths)**					
		**All Causes**	**Respiratory Diseases**	**COPD**	**Pneumonia and Influenza**	**Circulatory Diseases**	**Ischemic Heart Disease**
2004	—	578.1	106.1 (18.4)	86.8 (15.0)	14.4 (2.5)	186.1 (32.2)	48.1 (8.3)
2005	—	577.7	109.7 (19.0)	89.0 (15.4)	15.6 (2.7)	184.0 (31.9)	46.6 (8.1)
2006	—	429.4	75.0 (17.5)	58.4 (13.6)	12.5 (2.9)	137.3 (32.0)	28.7 (6.7)
2007	—	507.2	87.9 (17.3)	68.7 (13.5)	15.3 (3.0)	168.6 (33.2)	39.8 (7.8)
2008	—	568.9	101.6 (17.9)	80.2 (14.1)	17.8 (3.1)	201.6 (35.4)	49.8 (8.8)
2009	—	586.2	102.4 (17.5)	82.7 (14.1)	16.0 (2.7)	214.8 (36.7)	55.1 (9.4)
2010	—	571.9	95.9 (16.8)	75.8 (13.3)	16.1 (2.8)	211.1 (36.9)	57.1 (10.0)
Mean	—	543.9	96.6 (17.8)	77.2 (14.2)	15.2 (2.8)	185.5 (34.1)	46.3 (8.5)

Mortality and population data were obtained from 12 Disease Surveillance Point (DSP) sites in Guangdong, Guangxi, Fujian, and Hainan provinces of China; COPD: Chronic obstructive pulmonary disease; Age standardized rates were calculated using the mean age-specific population size during 2004–2010.

### 3.2. Mortality by Sex and Age Group

**Figure 2:**
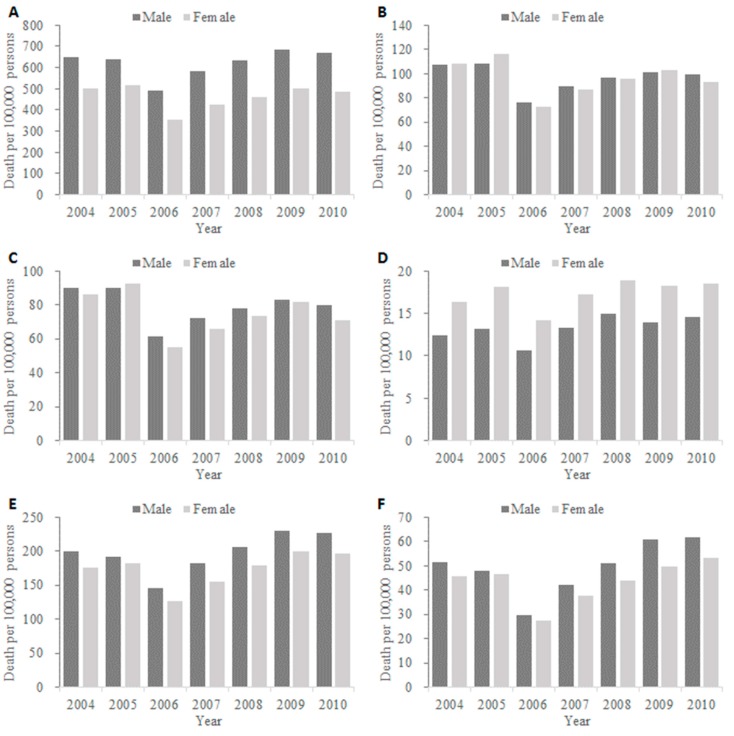
Average mortality rates by death cause and sex in Southern China, 2004–2010. (**A**) All-cause; (**B**) Respiratory diseases; (**C**) COPD; (**D**) Pneumonia and influenza; (**E**) Circulatory diseases; (**F**) Ischemic heart disease.

Table 2 provides the average deaths rates in the male and female population by age. The all-age and age-specific death rates show sex differences that were higher in the male population for all-cause, especially for the noted differences with twice the number in male in adults aged 15–64 years (341.0 *vs.* 147.4 per 100,000) and elderly aged 65–74 years (3252.0 *vs.* 1632.8 per 100,000), as well as for COPD and circulatory diseases (including ischemic heart disease) (Figure 2, panel A,C,E and F). Interestingly, the pneumonia and influenza death rates present a different sex distribution (Figure 2, panel D), which was very similar in children, and higher in male aged 16–64 years and 65–74 years but was opposite in elderly aged ≥75 years.

**Figure 3:**
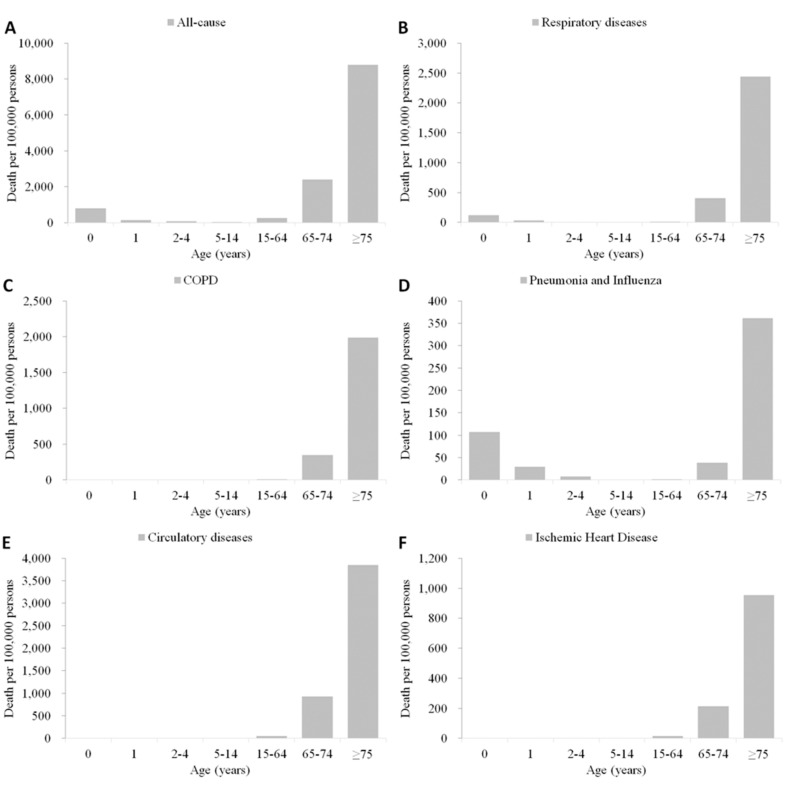
Average mortality rates by death cause and age group in Southern China, 2004–2010. (**A**) All-cause; (**B**) Respiratory diseases; (**C**) COPD; (**D**) Pneumonia and influenza; (**E**) Circulatory diseases; (**F**) Ischemic heart disease.

For age distribution of mortality in southern China, death rates of all-cause shows a common J-shape curve, in which the elderly showed the highest rates, the young children aged ≤5 years showed moderate while the school-age children and working-age adults showed the lowest rates (Table 2 and Figure 3, panel A). Death rates of respiratory diseases (Figure 3, panel B), pneumonia and influenza (Figure 3, panel D), had a similar J-shape curve in young children, and the ratio was relatively higher in respiratory diseases that that in pneumonia and influenza. Limited deaths were coded as COPD and circulatory diseases in the children group aged less than 15 years, and with these death causes, the death rates increased with age (Figure 3, panel C,E and F).

**Table 2:** Average death rates (per 100,000 people) by age and sex in Southern China, 2004–2010.

Age Group	Death Rates per 100,000 People (95% CI)					
	All Causes		Respiratory Diseases		COPD	
	Male	Female	Male	Female	Male	Female
0–11 months	825.8 (625.3, 1026.4)	770.8 (469.3, 1072.2)	119.2 (87.7, 150.6)	120.1 (64.8, 175.3)	1.3 (−1.1, 3.7)	1.1 (−0.9, 3.1)
1 year	148.1 (117.3, 178.9)	125.8 (98.2, 153.4)	33.1 (18.8, 47.5)	31.2 (10.6, 51.9)	0.3 (−0.5, 1.2)	0 (0, 0)
2–4 years	75.4 (65.4, 85.4)	62.0 (50.4, 73.5)	8.7 (6.2, 11.2)	9.1 (3.5, 14.6)	0 (0, 0)	0 (0, 0)
5–14 years	34.8 (30.0, 39.6)	20.5 (17.6, 23.4)	1.2 (0.8, 1.6)	1.1 (0.7, 1.5)	0 (−0.1, 0.2)	0.1 (−0.1, 0.3)
15–64 years	341.0 (315.1, 367.0)	147.4 (128.4, 166.3)	15.8 (12.8, 18.9)	8.2 (5.8, 10.7)	11.8 (9, 14.6)	6.1 (3.7, 8.4)
65–74 years	3252.0 (2894.0, 3610.0)	1632.8 (1389.2, 1876.4)	546.2 (443.8, 648.6)	277.5 (212.7, 342.3)	476.3 (380, 572.5)	238.7 (179.5, 298.0)
≥75 years	10,759.8 (9290.6, 12,229.1)	7572.0 (6418.4, 8725.6)	2912.0 (2592.2, 3231.8)	2149.2 (1822.5, 2476.0)	2459.9 (2198.1, 2721.6)	1696.3 (1427.5, 1965.1)
All-age	620.5 (369.6, 871.4)	462.9 (199.0, 726.8)	96.8 (31.7, 162.0)	96.4 (22.9, 169.8)	79.2 (24.4, 134.1)	75.0 (17.0, 132.9)
**Age Group**	**Pneumonia and Influenza**		**Circulatory Diseases**		**Ischemic Heart Disease**	
	**Male**	**Female**	**Male**	**Female**	**Male**	**Female**
0–11 months	107.3 (78.3, 136.2)	107.8 (58.4, 157.2)	13.5 (3.5, 23.6)	8.9 (4.3, 13.5)	0.3 (−0.5, 1.1)	0.7 (−1.1, 2.6)
1 year	30.4 (17.2, 43.7)	28.8 (9.1, 48.6)	4.1 (0.9, 7.2)	2.4 (0.2, 4.6)	0 (0, 0)	0 (0, 0)
2–4 years	7.7 (4.9, 10.4)	7.9 (2.7, 13.1)	0.6 (0, 1.1)	0.5 (0.1, 1)	0 (0, 0)	0 (0, 0)
5–14 years	1.0 (0.7, 1.3)	0.8 (0.6, 1)	1 (0.6, 1.3)	0.5 (0.3, 0.7)	0 (−0.1, 0.2)	0 (0, 0.1)
15–64 years	2.6 (2.0, 3.1)	1.5 (1.1, 1.9)	68.3 (62.2, 74.3)	34.6 (30.1, 39)	20.3 (17, 23.7)	8.7 (6.8, 10.7)
65–74 years	48.3 (40.7, 55.9)	29.9 (24.6, 35.1)	1226.5 (1102.3, 1350.6)	666.7 (567.6, 765.8)	278.8 (231.9, 325.8)	153.5 (125.5, 181.5)
≥75 years	347.2 (276.1, 418.2)	369.8 (298.9, 440.8)	4682.4 (3821.4, 5543.3)	3334.9 (2674.8, 3995.1)	1117.9 (850.5, 1385.3)	850.9 (643.8, 1058)
All-age	13.3 (5.1, 21.4)	17.4 (4.5, 30.2)	197.1 (88.4, 305.7)	173.3 (57.1, 289.5)	49.1 (23.7, 74.5)	43.3 (13.8, 72.7)

Mortality and population data were obtained from 12 Disease Surveillance Point (DSP) sites in Guangdong, Guangxi, Fujian, and Hainan province of China; COPD: Chronic obstructive pulmonary disease.

### 3.3. Temporal Trend of Mortality

**Figure 4:**
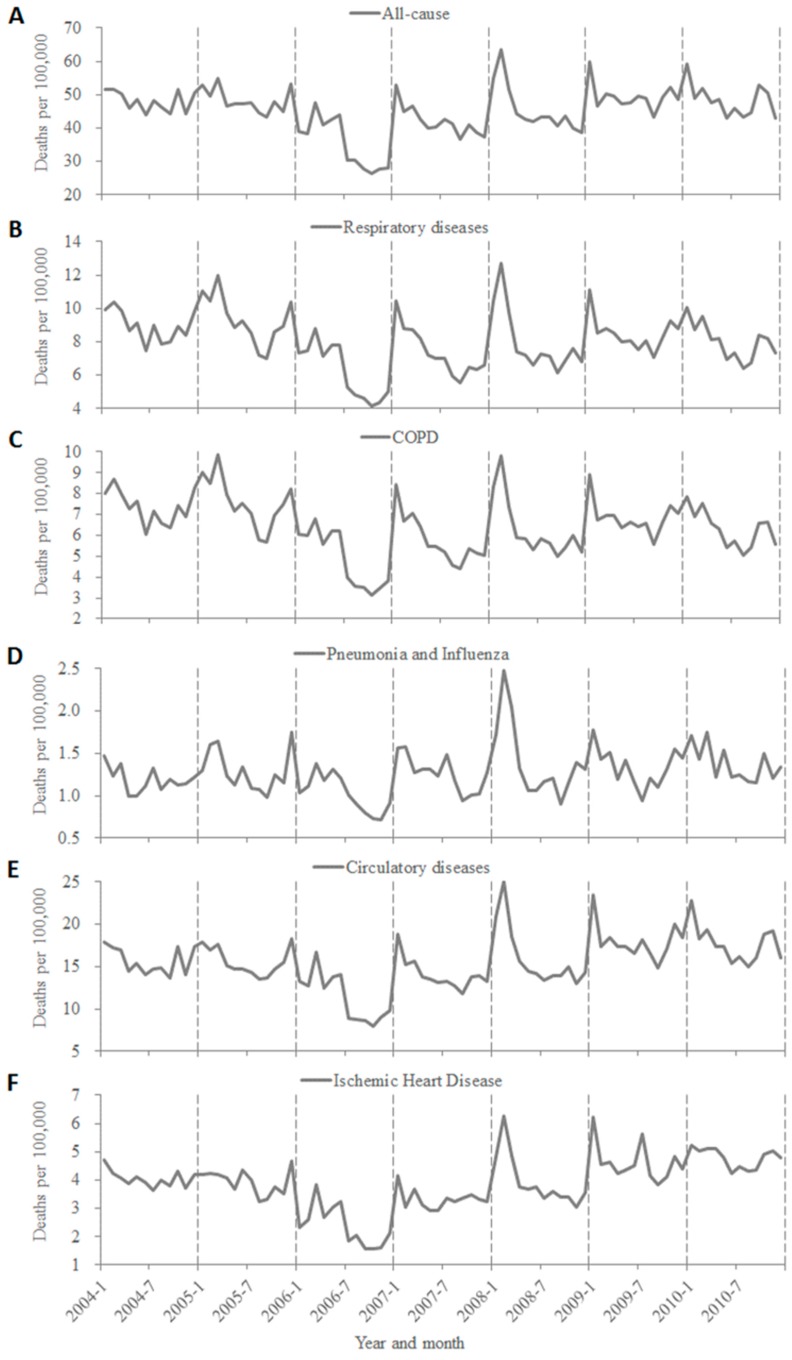
Monthly deaths per 100,000 people by underlying causes in Southern China, 2004–2010. (**A**) All-cause; (**B**) Respiratory diseases; (**C**) COPD; (**D**) Pneumonia and influenza; (**E**) Circulatory diseases; (**F**) Ischemic heart disease.

During the 7-year study period, all categories of death peaked in winter months (December to February), with year-to-year variations in peaking time (Figure 4). In general, annual death rates of all categories were relatively constant throughout the study period, except for 2006, in which fewer deaths were registered especially in its second half-year. A clear seasonal pattern of winter peak was seen for the deaths of all categories during 2007–2010, with a much higher death rate in 2007–2008 winter season.

## 4. Discussion and Conclusions

Our study provides sex- and age-specific mortality data of all-cause and specific categories of respiratory or circulatory diseases in four subtropical and tropical southernmost provinces, based on a unique and representative surveillance system of mortality. We found that annual death rates were 543.9 (range: 423.6 to 593.6) per 100,000 people for the 12 selected sites during 2004–2010, whereas over half the deaths were coded as respiratory and circulatory diseases. The crude death rates in 2010 (580.0 per 100,000 population) were similar to China’s national level (607 per 100,000 population) as well as to those of Argentina, Mexico, Turkey, and other middle-income countries, reported from the Global Burden of Disease Study 2010 [14]. The death rates of specific categories of respiratory or circulatory diseases are comparable or incomparable, e.g., COPD (77.2 per 100,000 in our study *vs.* 70.6 per 100,000 population for a national estimate in the Global Burden of Disease Study 2010), circulatory diseases (185.5 *vs.* 230.8 per 100,000), and ischemic heart diseases (46.3 *vs.* 70.1 per 100,000) [14].

However, as a crude rate, it should be noted that the mortality data for 2006–2010 were not adjusted for the underreporting rates, which attributed to a drop of death rates from 2006 compared with the previous two years. After 2006, the completeness and quality of mortality registration improved, and the all-cause mortality rates were consistent during 2007–2010. Two approaches of controlling and monitoring the quality of mortality data were used within the DSP system. The first was an internal procedural check system developed based on the online information management system, which evaluated timeliness and completeness of death registration, and the accuracy of data entry. This check method could detect errors, which could be corrected through re-enquiry; thereby improving the validity of the datasets of vital registration. The second method was to evaluate the datasets using statistical techniques and measures. Yang *et al*. [2] evaluated the completeness and accuracy of data of death registration in the DSP in 1999 using the standard United Nations Age Sex Accuracy Index [15], and the result suggested that the age-sex mortality data were accurate. Additionally, re-surveys based on “capture-mark-recapture” methods were conducted every three years in the 1990s, and each survey covered a sample of 5000 households in each province. Surveys conducted in 1992, 1995, and 1998 found that the completeness of deaths registration for infants and young children was lower than that of adult deaths [16,17]. It is worth noting that the rate of under-assessment was comparable in both urban and rural areas, and there was no improvement in the reporting completeness in the three successive surveys. As mentioned above, a national survey in 2006 checked the quality of reported data during 2004–2005 [12]. The recent under-reporting survey of mortality registration in China conducted in 2009 suggested that the average underreporting rate for the 161 DSP sites was estimated to be 17.4% in 2006–2008, and the rates in the middle and west regions were higher than in the east (including the four southernmost provinces in this study), which were 19.27%, 18.15%, and 15.46%, respectively [18]. We believe the underreporting rates of 12 selected sites were relatively lower than the east region defined in the 2009 survey, as we excluded seven sites where mortality data were not reliable after data quality evaluation.

Death rates of all-cause, COPD and circulatory diseases showed noted sex differences, with higher rates in the male population for all-age and specific age groups, which may be due to the significant differences of sex-specific prevalence of these diseases or conditions (such as COPD) and related risk factors (such as smoking). A large, population-based survey on the COPD prevalence of residents 40 years of age or older in seven provinces of China estimated the overall COPD prevalence was 12.4% in male and 5.1% in female [19]. Our findings of age and sex distribution of circulatory disease deaths were consistent with the differences in mortality rates of cardiovascular diseases in Finland, with significant higher rates in men, especially for the age of 15–74 years [20]. The results were also comparable with the age and sex distribution in many other areas, including the United States [21] and Hong Kong SAR [22]. The sex differences of mortality could also attribute to sex biases in both incidences and severity of some infectious conditions, indicating the influence of differential levels of sex hormones throughout different stages of life [7]. Pneumonia and influenza death, a more specific indicator for the severity of influenza viruses and other respiratory pathogens that could cause lower respiratory tract infections, had a distinct age distribution, with higher death rates in children, compared with limited deaths coded as circulatory diseases and another respiratory disease, COPD. This hints that infectious diseases posed higher risks of death outcomes in young children, and thereby provides complementary evidence to use mortality data to estimate excess mortality burden of influenza and other respiratory infections.

Furthermore, a clear seasonality pattern with peaks was seen in all-cause deaths and the specific categories. The phenomenon that mortality peaks occurred in the coldest months and waves in the hottest months is supposed to be correlated to climate factors, especially in extreme temperatures. Meanwhile, those pathogen factors with similar seasonality could also partially account for the temporal changes in mortality patterns of the study sites. However, it is necessary to consider carefully when drawing a causal relation between the mortality outcome and the factors mentioned above, as multiple factors and complex mechanisms can affect the mortality pattern of the population. For example, a recent study reported influenza A viruses peaked in April-June in these southernmost provinces, while influenza B activity predominated in colder months, and the activity of influenza B viruses coincided with the peak of deaths of all-cause and each category [5]. Therefore, the roles of influenza viruses and other potential factors in driving the mortality trends await to be further discussed and quantified. Interestingly, the higher death rates of each category observed in the winter season of 2007–2008, may be attributed to co-circulation of influenza B and A(H3N2) viruses, which were shown in two recent influenza-associated mortality studies [3,4], as well as the specific pneumonia and influenza deaths, or related to climate factors or activities of other pathogens in that period. There was one limitation in that we only used descriptive analysis on the monthly and seasonal distributions, but we will choose adequate statistical methods to test the found seasonality trends in further studies.

In conclusion, the present study reported intriguing sex and age differences in all-cause deaths and specific categories of respiratory or circulatory diseases in southernmost China. Our work highlights a relatively higher risk of severe death outcomes in the male population and specific age groups, which has practical implications for planning public health policy of intervention in this region, such as considering target population for an intervention when resources are limited. Further studies should be undertaken to explore the causes of this phenomenon and to quantify the balance between mortality patterns and their potential drivers, including population, climatic, and pathogenic factors.
